# Hypoglycemia frequency and treatment satisfaction in patients receiving insulin analogues for treatment of type 1 diabetes mellitus

**DOI:** 10.20945/2359-3997000000332

**Published:** 2021-02-25

**Authors:** Gabriela Berlanda, Gabriela H. Telo, Bárbara Côrrea Krug, Rafael Selbach Scheffel, Bruna Pasinato, Fernando Iorra, João Gabbardo dos Reis, Paulo Dornelles Picon, Beatriz D. Schaan

**Affiliations:** 1 Universidade Federal do Rio Grande do Sul Programa de Pós-Graduação em Endocrinologia Porto Alegre RS Brasil Programa de Pós-Graduação em Endocrinologia, Universidade Federal do Rio Grande do Sul (UFRGS), Porto Alegre, RS, Brasil.; 2 Hospital de Clínicas de Porto Alegre Porto Alegre RS Brasil Hospital de Clínicas de Porto Alegre, Porto Alegre, RS, Brasil.; 3 Universidade Católica do Rio Grande do Sul Escola de Medicina da Pontifícia Departamento de Medicina Interna Porto Alegre RS Brasil Departamento de Medicina Interna, Escola de Medicina da Pontifícia Universidade Católica do Rio Grande do Sul, Porto Alegre, RS, Brasil.; 4 Secretaria Estadual da Saúde do Rio Grande do Sul Porto Alegre RS Brasil Secretaria Estadual da Saúde do Rio Grande do Sul, Porto Alegre, RS, Brasil.; 5 Universidade Federal do Rio Grande do Sul Porto Alegre RS Brasil Universidade Federal do Rio Grande do Sul (UFRGS), Porto Alegre, RS, Brasil.; 6 Comitê de Contingência de Combate ao COVID-19 São Paulo SP Brasil Coordenação Executiva do Comitê de Contingência de Combate ao COVID-19 do Governo do Estado de São Paulo, SP, Brasil.

**Keywords:** Type 1 diabetes, hypoglycemia, treatment satisfaction, insulin analogues

## Abstract

**Objective:**

The aim of this study was to evaluate the frequency of hypoglycemia and the treatment satisfaction in patients with type 1 diabetes (T1D) using insulin analogues.

**Subjects and methods:**

This observational retrospective study included 516 adult patients with T1D from 38 cities in Southern Brazil. Demographics and clinical data were collected using a self-report questionnaire. Hypoglycemia was defined as an event based on either symptoms or self-monitored blood glucose < 70 mg/dL. Treatment satisfaction was evaluated using the Diabetes Treatment Satisfaction Questionnaire status version (DTSQs) and with a specific question with scores ranging from 0–10. Common mental disorders were assessed using the General Health Questionnaire (GHQ-12).

**Results:**

Overall, the mean age was 38 ± 14 years and 52% of the participants were women. The median diabetes duration was 18 years. The scores for insulin analogue treatment satisfaction were higher than those for previous treatments. DTSQ scores had a median value of 32 (interquartile range 29–35) and remained unchanged over time. The percentage of patients with hypoglycemia (including severe and nocturnal) was comparable across groups divided according to duration of use of insulin analogues. Most patients (n=395, 77%) screened positive for common mental disorders.

**Conclusions:**

Patient satisfaction with insulin analogue treatment was high and remained unchanged with time. Episodes of hypoglycemia also remained unchanged over time among patients using insulin analogues.

## INTRODUCTION

Type 1 diabetes mellitus is a chronic and progressive disease with an increasing incidence over the past decades. Estimates project that 5–10% of all 12 million patients with diabetes in Brazil have type 1 diabetes (1). The increased morbidity and mortality due to microvascular and macrovascular complications related to this disease result in reduced quality of life and life expectancy (2,3).

The Diabetes Control and Complications Trial (DCCT) has shown that strict glycemic control in patients with diabetes significantly decreases the risk of chronic complications (4). After the trial, multiple-dose insulin or insulin pumps have become the recommended treatment for these patients. However, achieving the recommended reduction in glycated hemoglobin (HbA1c) levels is very difficult due to multiple factors, including a high frequency of hypoglycemia (4–6).

Because of their pharmacological profile, insulin analogues can better mimic endogenous insulin production compared with human insulin, thus contributing to a decreased frequency of hypoglycemia and improved treatment satisfaction (7). Some studies have suggested that insulin analogues are associated with greater patient satisfaction with treatment, regardless of clinical outcomes (8). However, these concepts are still the subject of debate, with disagreements between protocols and guidelines worldwide, since most studies in which they are based have low methodological quality, are not blinded, and have been funded by pharmaceutical industry with potential overestimation of the benefits of the product/intervention evaluated.

Another limitation in interpreting the results of studies that have associated the use of insulin analogues with better patient satisfaction with treatment is that other parameters such as depression and anxiety can also impact patient satisfaction (9). Indeed, common mental disorders have been associated with low adherence to treatment and poor glucose control in patients with type 1 diabetes mellitus (10,11).

In addition to the concerns regarding the potential advantages of therapy with insulin analogues, it is unclear whether patient satisfaction with this specific therapy could reduce over time, as observed with other interventions in chronic diseases (12). We hypothesized that insulin analogues are associated with lower rates of hypoglycemia, greater dose flexibility, and higher treatment satisfaction among patients with type 1 diabetes mellitus. Based on that, the purpose of this study was to evaluate the frequency of hypoglycemia and treatment satisfaction in patients with type 1 diabetes mellitus treated with insulin analogues after introduction of these drugs in the public health system in Southern Brazil.

## SUBJECTS AND METHODS

### Design and procedures

This was an observational study carried out from April 2016 to December 2017. The protocol of the study was approved by the research ethics committee of *Hospital de Clínicas de Porto Alegre* (*Certificado de Apresentação para Apreciação* Ética –CAAE-1.283.728).

The primary outcomes were the frequency of hypoglycemia and treatment satisfaction of patients with type 1 diabetes mellitus after starting treatment with insulin analogues provided by the government, seeking possible predictors of greater satisfaction and fewer hypoglycemic events. A secondary outcome was the impact of the duration of treatment with insulin analogues on the primary outcomes.

The public health system in the state of Rio Grande do Sul is geographically divided into 19 regional health coordinating units. These 19 units comprehend different numbers of municipalities, totaling 498 in the entire state. To determine the locations for data collection, we first selected 19 municipalities to represent each coordinating unit. Other 21 municipalities were randomly selected to complete the number of patients necessary to represent the regional health coordination units that received insulin analogues in the state.

Patients were invited to participate in the study upon their arrival at the pharmacy to pick up short- or long-acting insulin analogues dispensed by administrative or judicial procedures. A written informed consent was signed by the patients included in the study or their legal guardians.

### Study population

The eligible population comprised patients aged 18 years or more, with type 1 diabetes mellitus treated with short-acting (including insulin lispro, aspart, and glulisine) and/or long-acting (including insulin glargine, detemir, and degludec) insulin analogues supplied by the Health Secretariat of Rio Grande do Sul (SES-RS) via an administrative or judicial procedure. Patients with impaired cognition or communication barriers were excluded.

To receive insulin analogues via an administrative procedure, a patient is required to have certain inclusion criteria, *i.e.*, type 1 diabetes mellitus, use of human insulin for at least 6 months, HbA1c level < 12%, at least two severe hypoglycemic events over 6 months, and follow-up with an endocrinologist for at least 6 months. Patients who did not meet all the criteria to receive insulin analogue via an administrative procedure appealed to the judiciary to receive the medication via a judicial procedure.

### Measures

Clinical and sociodemographic characteristics were obtained using a self-report questionnaire. For the hypoglycemic outcomes, the questionnaire referred to the month before study enrollment. Hypoglycemia was defined as an event based on either symptoms or self-monitored blood glucose < 70 mg/dL. Severe hypoglycemia was defined according to the American Diabetes Association as any hypoglycemic event requiring assistance of another person to actively administer carbohydrate, glucagon, or other resuscitative actions (13). Nocturnal hypoglycemia was considered when the hypoglycemic event occurred between bedtime and morning rise (14).

Treatment satisfaction was analyzed using the Portuguese version of the Diabetes Treatment Satisfaction Questionnaire status version (DTSQs), an 8-item inventory assessing therapy for type 1 diabetes mellitus in the previous weeks. This survey measures general satisfaction, adequacy, flexibility, diabetes understanding, and willingness to recommend the current treatment to other people and maintain such treatment (15). Each item is measured on a seven-point Likert scale ranging from 0 (very dissatisfied) to 6 (very satisfied). The DTSQs items two and three evaluate glucose control (perceived hyperglycemia and perceived hypoglycemia). These items are ranked differently, *i.e.*, 0 represents “never”, and 6 represents “most of the time”. Scores for all DTSQs items except for items two and three were used for the total DTSQs score, which ranged from 0 to 36. Higher scores indicated greater treatment satisfaction.

For mental health screening, the participants filled out the 12-item General Health Questionnaire (GHQ-12) translated into Brazilian Portuguese and validated for the Brazilian population (16). This self-report screening questionnaire assesses nonpsychotic symptoms of mental health in community settings by verifying whether the individual recently experienced specific symptoms or behaviors on a four-point Likert scale ranging between 1 to 4 for each item. A score ≥ 3 indicates the occurrence of common mental disorders (17).

Experience with the current and previous treatments was also evaluated using a specific question with scores ranging from 0 to 10, obtained through a self-report questionnaire. Other relevant survey information, including the patients’ weight (kg), height (cm), and insulin dispensing process (by administrative or judicial proceedings), were collected from the SES-RS computerized drug delivery system by a trained and authorized researcher.

### Data analysis

Considering an alpha error of 5% and a confidence interval range of 8%, the sample size calculation yielded 527 individuals.

Data were described using measures of central tendency (mean and median values) and dispersion (standard deviations and interquartile range [IQR]) for continuous variables and absolute numbers and proportions for categorical variables. Analysis of variance (ANOVA) was used for comparing numerical variables with normal distribution, and the Kruskal-Wallis test for data without normal distribution. The Mann-Whitney and Wilcoxon tests were used for paired samples, and the chi-square test was used to compare categorical variables. P values < 0.05 were considered statistically significant. All analyses were performed using the statistical software SPSS v.16.0 (IBM Corp., Armonk, NY, USA).

## RESULTS

### Demographics

From 4124 patients with type 1 diabetes mellitus older than 18 years receiving insulin analogues from SES-RS, we selected 566 potential participants. Of these, 50 were excluded for not meeting the inclusion criteria, yielding a final sample of 516 patients. The patients in the final sample belonged to 38 of the 40 municipalities included in the sample selection. The collection of data from some municipalities was difficult and we were unable to achieve 100% representation for each coordinating unit.

The participants in the study population had a mean age of 38 ± 14 years; 52% were women and 37% had a college degree. The mean body mass index (BMI) was 25 ± 5 kg/m^2^ (Table 1). The median duration of type 1 diabetes mellitus was 18 years (IQR 11–25 years). Overall, 431 patients (86%) used long-acting insulin analogues, and 458 patients (91%) used short-acting insulin analogues. Among the patients using short-acting insulin analogues, 105 (24%) received a flexible prandial insulin dose based on capillary blood glucose levels and carbohydrate counting. Insulin pumps and continuous glucose monitoring systems were not used by any of the patients. Among all 516 patients, 253 (49%) performed more than three blood glucose tests a day, 191 (37%) performed between two and three tests a day, and 72 (14%) performed at the most one test a day. When clinical and sociodemographic characteristics were analyzed according to the duration of use of insulin analogues (≤ 1 year, > 1 year, ≤ 5 years, and > 5 years), the group of patients using insulin analogues for ≤ 1 year had a mean age at diabetes diagnosis greater than the one using insulin analogues for > 5 years and a mean disease duration shorter than all other groups (Table 1). Regarding patients using long-acting insulin analogues, 293 (68%) received insulin from the SES-RS via an administrative procedure and 138 (32%) received it via a judicial procedure. Among patients using short-acting insulin analogues, 373 (81%) received insulin from the SES-RS via an administrative procedure, 81 (18%) received it via a judicial procedure, and some patients (1%) purchased their short-acting insulin analogues. Not all patients received both insulin types from SES-RS.

**Table 1:** Baseline patient and disease characteristics stratified according to the duration of use of insulin analogues

Characteristic	Overall study population	≤ 1 year	> 1 year and ≤ 5 years	> 5 years	P value
	(n=516)	(n=53)	(n=132)	(n=275)	
Age (years)	38 ± 14	37 ± 12	40 ± 13	38 ± 14	0.21
Sex (% women)	259 (52)	28 (52)	72 (55)	142 (52)	0.86
Ethnicity (% white)	446 (88)	44 (82)	114 (86)	207 (75)	0.05
School (% complete higher education)	189 (37)	20 (38)	42 (32)	113 (41)	0.18
Age at diagnosis (years)	17 (11-27)	22 (12-30)	20 (12-28)	15 (10-26)**	0.01
Diabetes duration (years)	18 (11-25)	13 (8-20)	18 (10-26)*	19 (12-27)**	0.01
Body mass index (kg/m^2^)	25 ± 5	25 ± 6	25 ± 5	24 ± 4	0.43
Rapid-acting insulin analogue use	458 (91)	45 (85)	123 (93)	251 (91)*	0.03
Long-acting insulin analogue use	431 (86)	45 (85)	118 (89)	237 (86)	0.52

Data are presented as mean ± standard deviation, median (interquartile range), or n (%). Analysis of variance (ANOVA) or Kruskal-Wallis test for continuous variables and chi-square tests for categorical variables, as indicated. P values

* p < 0.05 versus ≤ 1 year

** p < 0.01 versus ≤ 1 year.

### Hypoglycemia

**Figure 1:**
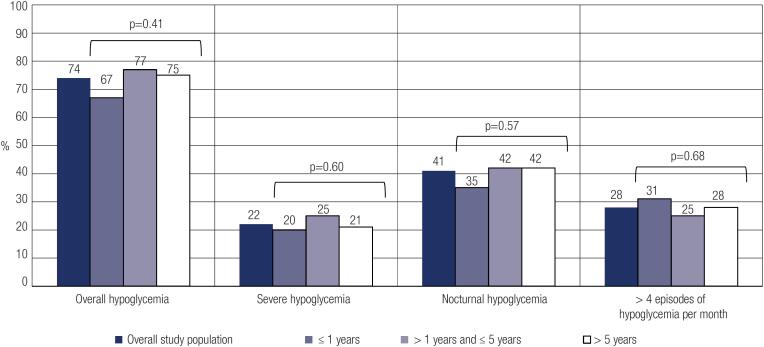
Percentages of patients with hypoglycemia according to duration of use of insulin analogues.

Among all patients included in the study, 350 (74%) reported hypoglycemia the month before data collection. Regarding the type of hypoglycemia experienced, 113 patients (22%) described severe hypoglycemia and 211 (41%) reported nocturnal hypoglycemia. A total of 144 patients (28%) reported more than four hypoglycemic events in the previous month. There were no differences among the percentages of patients reporting hypoglycemia when analyzed by groups divided according to duration of use of insulin analogues (Figure 1) or type of protocol for insulin analogue acquirement (administrative or judicial).

### Treatment satisfaction

The median DTSQs score in the overall sample was 32 (IQR 29–35) (Table 2). The median hyperglycemia perception score (DTSQs item two) was 3 (IQR 2–4) and the median hypoglycemia perception score (DTSQs item three) was 2 (IQR 1–3). No differences in treatment satisfaction were observed in relation to the duration of use of insulin analogues (Table 2) or between patients younger or older than 38 years (data not shown). When treatment satisfaction was assessed with the DTSQs among patients who received insulin analogues via administrative or judicial proceedings, no difference was found.

**Table 2:** Scores in the Diabetes Treatment Satisfaction Questionnaire (DTSQ) and General Health Questionnaire (GHQ-12) and in insulin experience according to duration of use of insulin analogues

Characteristic		Overall study population	≤ 1 year	> 1 year and ≤ 5 years	>5 years	P value
		(n=516)	(n=53)	(n=132)	(n=275)	
DTSQ, score						
	Treatment satisfaction	32 (29-35)	32 (29-34)	32 (30-35)	32 (29-34)	0.28
	Perceived frequency of hyperglycemia	3 (2-4)	3 (2-4)	3 (2-4)	3 (2-4)	0.44
	Perceived frequency of hypoglycemia	2 (1-3)	3 (1-4)	2 (1-3)	2 (1-3)	0.80
GHQ- 12						
	Screening CMD (≥ 3)	395 (77)	44 (85)	102 (77)	217 (80)	0.51

Data are presented as median (interquartile range). DTSQ total score, items 1, 4, 5, 6, 7, 8 (range 0–36, higher scores = greater satisfaction and lower scores = lower satisfaction). DTSQ items 1 and 2 (0 = never and 6 = most of the time). CMD: common mental disorders; score ≥3 = positive for CMD. Kruskal-Wallis test for continuous variables and chi-square tests for categorical variables. There were no statistically significant differences between groups.

The satisfaction score with the experience with current treatments (short- and long-acting insulin analogues) was obtained with a specific question with scores ranging from 0 to 10. The score obtained (10, IQR 9–10) was higher than the score obtained for previous treatments (NPH and/or regular human insulin), which was 5 (IQR 3–6, p < 0.001), reflecting increased satisfaction over time (p < 0.001). Patients without prior use of NPH or regular human insulin were not included in these analyses.

### Mental health

Most patients (n = 395, 77%) had a positive screening (score ≥ 3, GHQ-12) for common mental disorders (Table 2). The rates of patients with positive screening for common mental disorders did not vary according to duration of use of insulin analogues, between patients aged < or ≥ 38 years (p = 0.87), or between type of protocol for insulin analogue provision (administrative or judicial).

## DISCUSSION

Strict glucose control in type 1 diabetes mellitus reduces the risk of chronic disease complications at the expense of an increased number of hypoglycemic events (4,18). The use of insulin analogues reduces such events and increases patient adherence and satisfaction (19). In this pioneering study evaluating the satisfaction of patients with type 1 diabetes mellitus receiving insulin analogues determined by a pre-established procedure in Southern Brazil, we found a high incidence of severe hypoglycemia and greater treatment satisfaction in patients using insulin analogues compared with treatment satisfaction in the prior period when they received human insulin. Interestingly, these outcomes remained unchanged over time during use of insulin analogues.

Hypoglycemia is one of the main problems in patients with type 1 diabetes mellitus. These patients have an indeterminable number of episodes of asymptomatic hypoglycemia and an average of two events of symptomatic hypoglycemia per week (20). In the Hypoglycemia Assessment Tool (HAT) observational study, which included 27,585 patients across 24 countries, 83% of the patients with type 1 diabetes mellitus reported at least one hypoglycemic event a month, 40.6% reported nocturnal hypoglycemia, and 14.4% reported severe hypoglycemia (21). However, 25% of the patients in the HAT study in Brazil had severe hypoglycemia, which was comparable to our data (22%), while 54% of the patients had nocturnal hypoglycemia, which was higher than the rate found in our study (41%) (22). Although the rates of hypoglycemia were high in our study, they were possibly higher with the patients’ previous insulin treatment, prior to the initiation of insulin analogues, since hypoglycemia was one of the criteria for use of insulin analogues in our population. The excessive use of basal insulin in our study could have also contributed to the high rates of hypoglycemia in patients using insulin analogues. Since we did not evaluate the proportion of basal bolus insulin, we were unable to identify this contribution to the prevalence of hypoglycemia in our population. Moreover, we found that the percentage of patients with at least one hypoglycemic episode per month during treatment with insulin analogues was lower than that observed in the HAT study in Brazil (91.7%). These differences between studies may be explained by the methods used to assess hypoglycemia, types of insulin used, and occurrence of hypoglycemia unawareness, since many patients are asymptomatic with blood glucose readings below 70 mg/dL or are less likely to wake up when hypoglycemia occurs at night. The number of patients on insulin analogues in the HAT study in Brazil is unavailable, but it was certainly much lower than that in our study, since the type of insulin usually provided by the health care system in Brazil for patients with type 1 diabetes is NPH and regular human insulin.

An interesting finding of our study was that the rate of hypoglycemia remained unchanged over time in patients who already used insulin analogues. In contrast, data from the literature indicate that the risk of hypoglycemia increases markedly with the duration of the disease (23).

As a group with prior risk of severe hypoglycemia, our study patients who received insulin analogues via administrative proceedings abided to the requirement of the dispensing protocol, in which the presence of at least two events of severe hypoglycemia within 6 months was one of the criteria. These patients receive insulin free of charge from the state, but bear the expense of the needles that are necessary for insulin injection, thereby promoting, in many cases, excessive needle reuse (24).

Similar to previously described in other self-report studies, the patients in the present study identified the episodes of hypoglycemia by the occurrence of symptoms, blood glucose testing alone, or a combination of both (21,22). This approach is both a strength and a limitation of the present study, as the inclusion of hypoglycemic episodes based on blood tests may also identify asymptomatic hypoglycemia. Differences in health care and local economic conditions may also affect the patients’ access to education and blood glucose monitoring materials, which may affect the recording of hypoglycemic episodes. These factors, along with patient education and health care delivery, are also likely to affect the occurrence of hypoglycemia (21,25).

Another important finding of our study was that the patients were considerably more satisfied with the current diabetes treatment with insulin analogues compared with prior treatment with NPH and regular human insulin, as evaluated using a specific question with scores ranging from 0 to 10. This may be an important result since, according to the literature, treatment satisfaction is associated with better glucose control (26). Previous clinical studies have also reported greater satisfaction in patients with diabetes receiving treatment with insulin analogues compared with human insulin (7,19,27). A 28-week randomized clinical trial has shown a significant increase in the overall satisfaction (measured by the DTSQs) in patients with type 1 diabetes mellitus treated with insulin analogues compared with decreased satisfaction in patients treated with human insulin (score difference between treatments 1.83 points, p < 0.001) (19). When we analyzed patient satisfaction (DTSQs) according to the duration of use of insulin analogues, we observed no decrease in satisfaction over time, in contrast to observations from other interventions in chronic diseases, such as insulin pumps (12). Considering that the patients in our study received the treatment from the government without any cost, the fear of losing the treatment could be associated with a false increase in patient satisfaction. To minimize this problem, the written informed consent signed by the patients included the information that the analysis would be confidential to avoid bias due to fear that their access to insulin analogues could be interrupted if they were not completely satisfied with the treatment.

In our evaluation of the DTSQs per individual item, satisfaction was high in the six subscales of the questionnaire. Factors such as the similarity of insulin analogues to endogenous insulin with regard to pharmacokinetics, regimen complexity reduction, and dose frequency may have contributed to greater treatment satisfaction among our patients (28,29). The use of an application device (disposable pen) for insulin analogues can also be associated with increased patient satisfaction, since patients on human insulin in Brazil usually use syringes instead of pens. Previous studies have reported increased preference and treatment satisfaction along with improved quality of life with pen devices compared with vial and syringe (30,31). This is in line with a recent report showing improved glucose control in patients with type 2 diabetes with use of pens for insulin injections, although frequent medical visits and supply of blood glucose strips could also be responsible for this improvement (32).

Our study found a high rate of patients screening positive for common mental disorders (77%) compared with a previous study in 358 patients with diabetes, in which 29% screened positive for common mental disorders (33). However, that study also included individuals younger than 18 years and patients with type 2 diabetes, different than our study, which included only adult patients with type 1 diabetes. Diabetes is one of the most psychologically demanding chronic diseases, as it requires daily drug administration and strict adherence to drugs, diet, and physical activity (34). Considering common mental disorders associated with low adherence to treatment and poor glycemic control in patients with type 1 diabetes mellitus, the integration of mental health screening and care into routine diabetes care could potentially improve glycemic control in these patients (10).

On analysis stratified by duration of use of insulin analogues, patients using insulin analogues for less than 1 year were older than those using insulin analogues for more than 5 years. This finding of patients newly enrolled in the protocol for the acquirement of insulin analogues being older at diagnosis may be related to a more stringent technical criteria for access to insulin analogues implemented after updates in the SES-RS dispensing protocol.

Limitations of the present study include the fact that patients who are dissatisfied with the therapy or with the paperwork involved for acquiring insulin analogues in Brazil have the option to switch back to human insulin, which is also offered free of charge. Other limitations include the observational and retrospective design of the study, and the use of self-report data (which may not have reflected accurately the current blood glucose level of the participants in cases of hypoglycemia), and the absence of measurements of glycemic control (*e.g.*, HbA1c), which were not available in the SES-RS computerized drug delivery system. Also, we did not evaluate if hypoglycemia unawareness could have influenced our results. However, the characteristics of our study enabled the inclusion of more patients, driving significant observations in regard to the real-life impact of patient satisfaction with treatment. The sample represented more than 50% of most regional health coordinating units, and due to factors including temporary shortages of insulin analogues and difficulty of applying the questionnaire in some cities, did not include the entire planned sample but was overall representative of the population.

In conclusion, despite high rates of hypoglycemia and positive screening for common mental disorders, patients with type 1 diabetes mellitus using insulin analogues maintained great satisfaction with their diabetes treatment, which remained unchanged in the long term, unlike reports from other interventions in chronic diseases. Due to our study design, we were unable to confirm that the hypoglycemia rates were higher with treatment received prior to insulin analogues, as hypothesized. Observational studies, such as the present one, are essential since they are conducted in a real-world environment and provide valuable data regarding the use of a drug in clinical practice without the strict supervision of a randomized controlled trial. Additional studies and analyses are needed to investigate in each health macroregion of the state a potential association between hypoglycemia and conventional predictive factors of hypoglycemia including ethnic, cultural, and health-related factors.
